# A Qualitative Approach on Motives and Aspects of Risks in Freeriding

**DOI:** 10.3389/fpsyg.2017.01998

**Published:** 2017-11-14

**Authors:** Anika Frühauf, Will A. S. Hardy, Daniel Pfoestl, Franz-Georg Hoellen, Martin Kopp

**Affiliations:** ^1^Department of Sport Science, University of Innsbruck, Innsbruck, Austria; ^2^Institute for the Psychology of Elite Performance, School of Sport, Health, and Exercise Sciences, Bangor University, Bangor, United Kingdom

**Keywords:** high-risk sport, extreme sport, skiing, risk-taking, risk-management

## Abstract

Recent research has shown that there are multiple motives for participation in high-risk sport; however these results have come from studies that consider a number of different sports. Therefore, the aim of the present study was to better understand the motives and risk-related aspects of freeriding, using a qualitative approach. Semi-structured interviews were conducted with 40 professional and semi-professional freeride skiers and snowboarders. All freeriders were highly experienced, of different age (19–44 years; 27.5 ± 4.5 years), gender (female = 13), and profession (professional athletes = 11). Analyses were done using MAXQDA software following a code theme approach. Mixed methods analyses using χ^2^-tests were computed for age (<25 years ≥) and gender (female/male) on motives and risk factors. Five emerging themes were found, namely Challenge (*n* = 36), Friends (*n* = 31), Nature (*n* = 27), Balance (*n* = 26), and Freedom (*n* = 26). A sixth theme Habit (*n* = 13) was allocated as a subtheme due to minor responses. With regard to risk management, participants decided upon a risk calculation strategy which included multiple factors (e.g., planning, conditions, current situation, knowledge, and experience). Trusting in one's own abilities, avoiding negative fear and having trusted partners were among the risk factors. Deliberately seeking out dangerous situations was not a motive. χ^2^-tests revealed no gender or age differences regarding aspects of risk (range of p-scores: *p* = 0.17–1.00) or motives (*p* = 0.16–1.00). Freeriding was shown to provide positive effects through participation. Some important factors seem to be motivational drivers for freeriders: challenging oneself, experiencing nature, contributing to deep friendships, freeriding as a counterbalance to everyday life and escape from restrictions. Contrary to prior research reports on sensation seeking, experienced freeriders do not search the risk; they seem to minimize it based on knowledge and experience. Analyses of the present data did not show any gender or age differences, which may suggest that experience plays a more important role in high-risk sports than age or gender. Future research should qualitatively investigate further terrain based activities and implement motives and risk-related factors in quantitative research.

## Introduction

Freeriding describes skiing and snowboarding in undeveloped natural spaces (Reynier et al., 2014), jumping from sheer cliffs (Brymer and Schweitzer, 2013a), and involves the risk of serious personal injury or even death, through avalanches or other natural hazards (Haegeli et al., 2012). The term *freeriding* is widely understood and accepted in snow sports and is included in the name of major competitions (e.g., “Freeride World Tour,” “Freeride World Qualifier”). Freeriding is also referred to as *out-of bounds skiing* (Haegeli et al., 2012) and *backcountry skiing* (Techel et al., 2015).

Freeriding is often categorized with other sports such as, BASE jumping, mountaineering, big wave surfing, etc., however, no common moniker is used in the literature for these sports; some researchers use the term *extreme sports* (Pain and Pain, 2005; Willig, 2008; Brymer and Schweitzer, 2013b,a), others choose *adventure sports* (Heggie and Caine, 2012; Kerr and Houge Mackenzie, 2012), and others *high-risk sports* (Castanier et al., 2010; Woodman et al., 2013; Barlow et al., 2015). In this paper we will use the term *high-risk sport*, which Breivik (1999, p. 10) defined as “all sports where you have to reckon with the possibility of serious injury or death as an inherent part of the activity,” thus, freeriding can be considered a high-risk sport.

Participation in high-risk sports is generally voluntary and participants usually know what the hazards involved are. Personal knowledge and technical skills allow participants to manage their exposure to these hazards within reason (Haegeli and Pröbstl-Haider, 2016). Traditionally researchers have suggested that all high-risk sports participants are sensation seekers (Zuckerman and Neeb, 1979; Llewellyn and Sanchez, 2008; Zuckerman, 2008). However, recent research in high-risk sport has shown that although this may hold true for some high-risk sport participants (e.g., skydivers) there are also a number of other behavioral and motivational antecedents of participation for others (e.g., emotion regulation for mountaineers) (Lafollie and Le Scanff, 2007; Llewellyn and Sanchez, 2008; Woodman et al., 2009, 2010; Castanier et al., 2010, 2011; Kerr and Houge Mackenzie, 2012; Barlow et al., 2013, 2015; Brymer and Schweitzer, 2013b; Ewert et al., 2013; Wiersma, 2014). Motives not only varied between activity types but also between experience level of participants (Ewert et al., 2013).

There is evidence in recent literature showing psychological benefits from participation in high-risk sports [e.g., affect regulation (Castanier et al., 2011) and emotion self-regulation (Cazenave et al., 2007; Woodman et al., 2009, 2010; Castanier et al., 2010, 2011; Barlow et al., 2013, 2015)]. Recent qualitative studies have shown further positive effects of participation in high-risk sports (Brymer, 2010). Brymer and Gray (2010) described how high-risk sport participants develop special relationships with nature. One possible explanation for this is that the vast majority of high-risk sports are performed outside in the natural environment, this type of environment has been shown to provide greater physiological and psychological benefits than exercising indoors (Ryan et al., 2010).

Other research suggests that high-risk sports allow participants to: experience freedom and thus, explore fundamental human values (Brymer and Schweitzer, 2013b); experience fear and anxiety which has transformational benefits (Brymer and Schweitzer, 2013a); and develop courage and humility (Brymer and Oades, 2009). These studies were carried out with participants older than 30 years because previous research has claimed that young people (16–25 years) search for opportunities to take deliberate risks across a range of activities and the researchers wanted to control for this (see Brymer and Schweitzer, 2013b).

From a behavioral perspective most of the research conducted in this field has focused on risk-taking behavior. Risk-taking seems to comprise two orthogonal factors; *deliberate risk-taking* (e.g., skiing an avalanche prone slope) and *precautionary behaviors* (e.g., wearing safety equipment, reading the avalanche forecast) (Woodman et al., 2013). Paquette et al. (2009) found that both recklessness and safety were risk-related aspects of participating in snowboarding. Research into personality types has shown differences in risk-taking in high-risk sport participants (Woodman et al., 2009; Castanier et al., 2010).

Willig (2008) challenged the longstanding view of health psychology that risk-taking is a sign of psychopathology and suggests that risk-taking in high-risk sports can have psychological benefits through four main themes: *context, challenge, suffering*, and *other people*. These themes were elicited from interviews with eight high-risk sport participants; three skydivers, two mountaineers, and two who practiced multiple high-risk sports. However, Barlow et al. (2013) showed that skydivers and mountaineers have different motives for participating in high-risk sport, which means that it might be important to consider separate groups of high risk sport participants.

Some of the most recent investigations into the motives for participation in high-risk, have used a qualitative, hermeneutic approach with a mixed sample of participants in high-risk sports (e.g., Willig, 2008; Brymer and Schweitzer, 2013b). However, more recently researcher have suggested that high-risk sport participants should not be considered as a homogenous group (e.g., Cazenave et al., 2007; Llewellyn and Sanchez, 2008; Woodman et al., 2009, 2010, 2013; Castanier et al., 2010, 2011; Barlow et al., 2013, 2015).

Freeriding is becoming increasingly popular (Pain and Pain, 2005) and is the fastest growing segment in the ski industry (Vargyas, 2016). Total numbers of participation are unobtainable and thus no mortality rates can be calculated (Brugger et al., 2013). Whereas, the total number of avalanche accidents seem stable (see Procter et al., 2014), the number of avalanche fatalities through backcountry recreationists (e.g., freeriders, snowshoers, snowmobilers) is growing in some areas and the majority of victims were male (Jekich et al., 2016). This may be an artifact of higher numbers of male participants (as shown in Leiter and Rheinberger, 2016). However, little is known about freeriders' motives for participation, nor is much known about participants' risk-taking during participation. Raue et al. (2015) described a difference in risk perception between experienced and less experienced freeriders during a ski tour. Experienced freeriders risk-perception remained stable, before, during, and after participation whereas, less experienced freeriders perceived the activity as less risky after participation than before. Raue et al. (2015) concluded that risk-perception is influenced by experience, emotion and by the current situation.

In high-risk domains there are a variety of hazards and the management of them is multi-faceted and the “boundary between acceptable and unacceptable is more gradual” (Haegeli et al., 2012); thus far attempts to identify people at risk in high-risk domains using questionnaires has been inconclusive. The present research sought to gain a better understanding of the motives for freeriding and participants perspectives on risk-related aspects. As earlier publications reported gender and age effects in high-risk samples, we were also interested in how, if at all, these variables are relevant in the population of freeriders.

## Methods

### Participants

In total, 40 freeride athletes were interviewed (*M*_*age*_ = 27.5 years, *SD* = 4.4; 11 snowboarders and 29 skiers). Participants were selected using a combination of purposive sampling strategies, namely criterion-based and maximum variation sampling (Patton, 1990). This approach ensured that participants had specific knowledge and experience of the phenomena of interest whilst allowing the analysis of age and gender effects (Sparkes and Smith, 2014). The primary criterion was that athletes received sponsorship for freeriding. Participants included 11 (two female) professionals (current or former Freeride World Tour athletes or riding for international movie productions); 22 athletes (10 female) who still participated in qualifying events for the Freeride World Tour; seven freeriders (one female) who had stopped competing but were still sponsored. To examine age and gender differences participants included 13 women and 27 men; 12 participants 18–25 years old, 20 participants 26–30 years old, and eight participants over 30 years old. Due to ethical issues no underage participants (<18 years) were included.

Participants were informed prior to the interviews verbally and they received an information sheet according to the ethical guidelines of Helsinki which was signed at the beginning of the interviews. Approval by the Board for Ethical Questions in Science of the University of Innsbruck in accordance with the Declaration of Helsinki, was given prior to the study (No. 47/2016, Date 21.12.2016).

### Procedure

A semi-structured interview was carried out with each participant. An interview guide was used to ensure that each participant was asked the same questions but also allowed them to talk freely about their experiences. The interview guide was developed by the research team based on existing research on high-risk sport participants. Three pilot interviews were conducted to assess the clarity of the questions and to familiarize the interviewers with the guide.

Interviews were carried out by three members of the research team. All bar two interviews were conducted in German, the other two were conducted in English as the participants felt more comfortable speaking English than German. All interviews were carried out one-to-one; 33 interviews were carried out face-to-face in a place of the participants' choosing; for seven interviews it was not possible due to geographic constraints to carry out face-to-face interviews. Therefore, they were carried out via Skype. The interviews lasted an approximately 30–40 min.

To build a rapport between the interviewer and participants, all interviews started with questions designed to the participant at ease (e.g., “How did you get involved in freeriding?”) and were followed with questions about the experiences that they have had in freeriding (e.g., “Could you tell me about your experience in freeriding?”). Given the effect of personality on risk-taking (Woodman et al., 2009; Castanier et al., 2010), the following section asked questions about the participants' personalities (e.g., “How would you describe yourself?”). In the following section, participants were asked questions about why they went freeriding and what they enjoyed about it (i.e., their motives). Participants were also asked to describe a particularly poignant experience (e.g., “Can you describe one memorable moment of freeriding?”). The final section asked participants questions about their risk-taking. They were asked to talk about their risk-taking in freeriding recreationally, when filming, when competing, as well as in everyday life (e.g., “Do you have different risk-taking behavior when competing?”). Specific probes were used to ask participants to compare their risk-taking with other peoples, including other freeriders and people who do not participate in high-risk sports (e.g., “What do you think of your risk-taking compared to your friends?”). To reduce the potential for the researchers introducing bias to the results, interviews were conducted by three different researchers. Existing guidelines for conducting qualitative research were adhered to, in a further effort to ensure rigor (Elliott et al., 1999).

### Analysis

**Figure 1:**
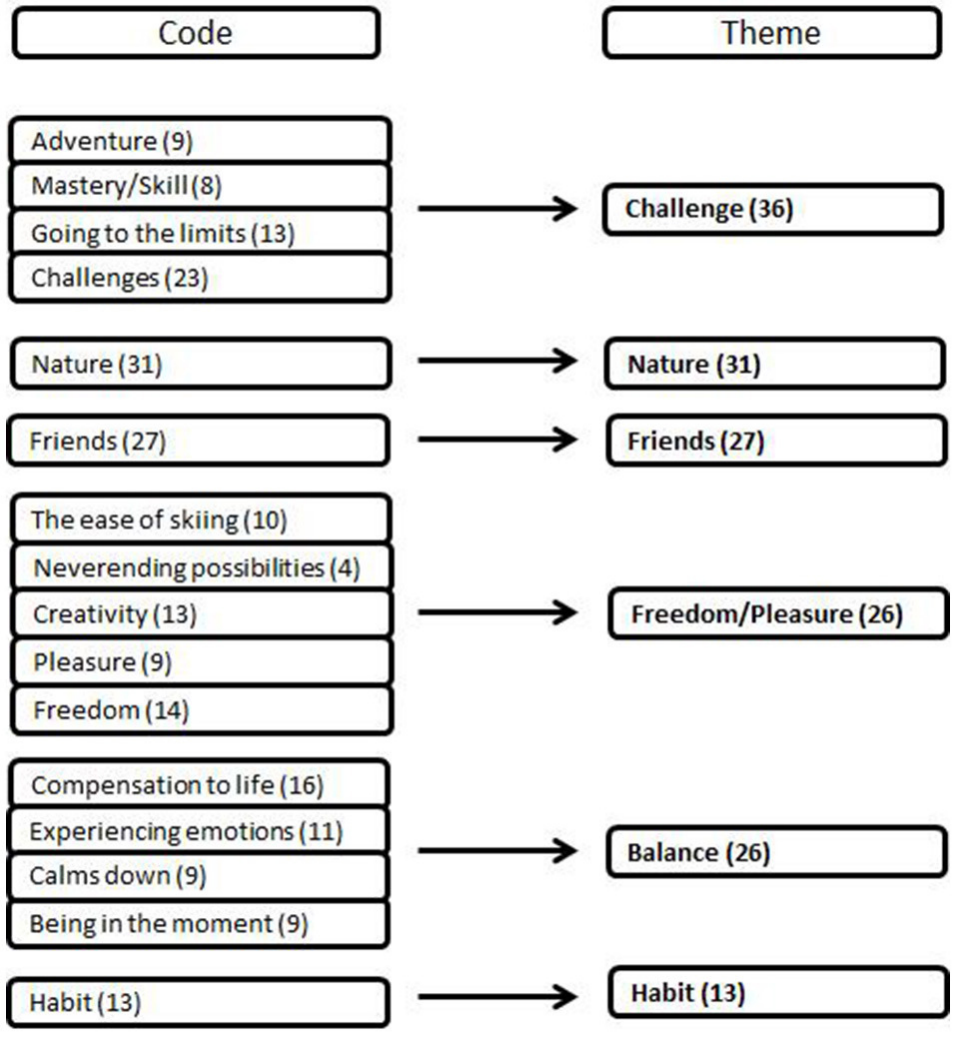
Codes and Themes of motives in freeriding. Codes were only accounted as one vote per person. Thus, the codes do not necessarily sum up to the according theme.

All interviews were transcribed verbatim and analyzed in the language that the interview was conducted in (German, *n* = 38; English, *n* = 2); transcription was carried out immediately after the interview to familiarize the research team with the data. Interesting phrases were highlighted and any non-verbal communications was noted. The data was then analyzed in several distinct stages using MAXQDA Software (*MAXQDA*, 1989–2017)^1^. Firstly, the first author read the transcripts a number of times to immerse themselves in the data. Secondly, they carried out an inductive hierarchical content analysis, raw data themes were given codes (e.g., “being in the moment”), when there was not a suitable existing code a new one was created; in total 35 codes were created. Whilst the analysis was conducted in both English and German, all codes and themes were named in English language to reduce translation bias. This procedure was repeated for all 40 interviews. In the next step of analysis, all interviews were cross-checked, ensuring that coding was consistent and accurately represented the data. Following this, similar codes were grouped into themes (*n* = 9; e.g., *being in the moment* and *experiences emotion* were grouped into the theme *balance*; see Figure 1). The final step was to confirm the codes and themes with the 5th author, who acted as a critical friend (Smith and Sparkes, 2009) and any disagreements were resolved by discussion. The only disagreement was about the name for the theme “Habit” and this was discussed until full consensus was reached. In addition to this, raw quotes have been presented in English, with the hope that the data will speak for itself and the voices of the participants might be heard. Gender and age differences in motives and aspects of risk were tested for using χ^2^-tests (alpha = 0.05) in SPSS 23.0 (2015).

## Results

### Participation

Almost all participants first experience of skiing was slope-skiing at an early age (2–5 years) with their families (*n* = 37). The majority of participants grew up in an alpine environment with an easy access to ski lifts (*n* = 34). Twenty three participants engaged in ski racing throughout their childhood (till 14–18 years) and stopped either due to an injury or motivational factors (e.g., “*too much pressure*”). Having already begun skiing, 11 participants started snowboarding during childhood. Participants started freeriding because of friends, family, education (ski instructor), new ski technology (freeride skis) or participation in freeride contests. Most participants (*n* = 30) said that their participation in freeriding was not due to one single event but it seemed to happen naturally.

### Motives for participation

Five main themes emerged from the code analysis of the data (Figure 1): Challenge (*n* = 36), Nature (*n* = 31), Friends (*n* = 27), Balance (*n* = 26), and Freedom/Pleasure (*n* = 26). A sixth theme, Habit (*n* = 13) was present in less than half of the interviews and thus, was a sub-theme. To test gender and age differences in motives, mixed methods analyses were applied. No statistically significant differences regarding gender (male/female) or age (below or above 25 years) were found (in all instances *p* > 0.1). Both male and female participants (Supplementary Table 1), as well as participants above or below 25 years (Supplementary Table 2), had similar motivations to go freeriding.

In the following section, themes will be explained and analyzed separately.

#### Challenge (36)

Experiencing the challenge of freeriding was the most frequently mentioned motivation; (90% of all participants; *n* = 36). Participants experienced *Challenge* in a number of ways: encountering new places with skis, exploring personal limits, experiencing mastery/skill, overcoming the challenges of environmental conditions, etc. (Figure 1). For some participants *Challenge* in freeriding was the opportunity to explore their personal limits. “Playing the mind game” (Female 9, 26 years, semi-professional) was one aspect of *Challenge*; being able to trust in one's own abilities when a mistake could be fatal,

I wouldn't say what we wanted to ski was harmless. I knew I could ski this due to my technical abilities, but it is pretty intense to know when I do something wrong—then it's over; you're not allowed to fall. It's this mind game: I am able to do this but a lot could go wrong—this matters in freeriding (Female 9, 26 years, semi-professional).

Participants also spoke about exploring and stretching their limits as an aspect of *Challenge*,

The thing is to work on your personal skills, improving your skiing and going to the limits of what is still possible. At first you think that's not possible, I can't ski that and then it works anyway and you have realized something which seemed impossible at first—this relocation of your limits (Male 21, 34 years, semi-professional).

Other participants stated that there were different ways of reaching their limits. Some people (*n* = 10; 25%) explicitly differentiated between trying to relocate their limits by improving their freestyle skills (e.g., jumping of higher cliffs, doing tricks above rocks and cliffs and being exposed in dangerous environments). When improving their freestyle skills, they were looking for a safe environment (in the backcountry) with relatively few natural hazards,

I have a lot of respect for external factors, like avalanches. I don't risk much there. In other parts I risk more. For example when it is about jumping off of cliffs, I feel at ease there…. But I always look at it rationally: when there is a “no-fall-zone” or a steep face where you can hurt yourself really badly, I don't do it, because I don't feel good there. I take less risk there and rather put more effort in it when I know if I crash, it won't happen too much (Male 23, 23 years, professional).

This participant explained how he chooses the cliffs/jumps based on environmental factors and how his risk-taking varies based on the severity of the consequences of falling. When it was about skiing in more exposed and dangerous terrain, they stated how they would never just try it. This was explained by another participant who described the difference between jumping about obstacles in a safe environment and freeriding (referred to as Big Mountain Snowboarding).

Street and slopestyle have other risks, there it is more about serious injuries. But in Big Mountain Snowboarding, when something goes wrong I'm dead; you can't really compare those risks. When it is about testing one's limits I can't just say “hey let's give it a try.” This would be reckless (Male 3, 30 years, semi-professional).

The majority of participants (*n* = 24) talked about how they only did things they knew they were capable of. The more experienced they were, the more they knew about their limits and where they “*leave the gray area”* how one participant said (Male 3 semi-professional, 30 years). Although participants' behavior changed as they grew older, the same change in behavior was mentioned by younger, highly experienced freeriders. This suggests changes in behavior were based on experience and not on age. Knowing one's limits and believing in their own abilities is part of a risk management strategy.

It is about experiencing my limits. This is a reason to go freeriding for most people. And because I played a lot in the backcountry I have a big repertoire of skills to get back to. That's why I can do stuff where a lot of people would have already backed off. My tool box is just bigger than those of others (Male 3, 30 years, semi-professional).

Participants (*n* = 23) felt that the challenge presented by freeriding was a positive thing and that it benefited them in their everyday lives.

I get to know my own limits. That teaches you a lot about yourself (Female 2, 24 years, semi-professional);

I always try to stay within my limits and not to overstrain myself. You get to know yourself in sports really well and you know what you are capable of, what you can dare to do and what not (Male 9, 23 years, semi-professional).

One participant highlighted a key difference between freeriding and other sports, many of the challenges in freeriding come from the natural environment rather than other competitors. Another participant reported how she preferred challenges posed by nature to those posed by other people and how this made freeriding different to other sports,

Freeriding is not man against man like in other sports; it's rather man against the mountain and the challenge with the nature—that's just something I need (Female 8, 27 years, semi-professional).

Challenges presented by the natural environment require participants to understand the risks involved in freeriding. Whilst all participants said that they knew about the dangers of freeriding, some believed that they could minimize the risks through high levels of preparation and thought that they were less likely to be affected by a certain situation (e.g., avalanches). Others said “the mountain knows no conscience” (Male 9 semi-professional, 34 years), meaning that outcomes are never certain, no matter how well-prepared you are. Participants spoke about avalanche deaths of people who were experienced, well-prepared and seen as “safe,” using this as evidence to suggest that there is always the chance of something happening,

Last year, a good friend of mine died in an avalanche. This was really intense. He was one person who I felt was reliable and safe; someone I really liked to go skiing with. The avalanche factor—you just can't eliminate it (Female 9, 26 years, semi-professional).

The challenges participants faced in freeriding were described as complex and unpredictable due to the dynamic environment. The challenge of minimizing the risk was also part of freeriding and whilst participants did not seek high-risk situations they acknowledge the effect that risk has on them,

I always want to minimize the risk…I try to make the risk as small as possible but I always know that there is a chance of something happening. And it can be falling from doing a drop or it can be a risk of avalanche coming down and so on…or just like skiing into the rocks… You always try to minimize the risk but there is always a chance that something can happen. And I guess that little chance is part of it; it gives you the adrenalin. Like if you would ski stuff that wouldn't give you any, when there was no risk in it, if you would have only stayed on the piste, yeah you can fall on the piste and break your leg as well but that is not exciting (Female 11, 27 years, semi-professional).

#### Nature (31)

Most participants (*n* = 31; 78%) stated that being outdoors in nature was a motivation to go freeriding. Freeriding allowed participants to explore and appreciate natural spaces in remote places that are only accessible on skis. Descriptions like “untouched nature,” “uniqueness,” “without manmade things” show how freeriding was about more than just being outside, but that it is also about being away from the built environment. Thus, being in nature, skiing outside of resorts, without lifts, and human disturbances was important to participants. When asked “Why do you go freeriding?” 20 participants simply said “nature.” Others elaborated on this,

For me personally it's the uniqueness of nature, it has always attracted me. Being able to ski this is a primary attraction (Male 19, 38 years professional);

And then nature… I love being outdoors. If you do a splitboard tour and you are in untouched natural environment, there is so much tranquility (Male 4, 31 years, semi-professional);

“Being out there without interferences, without manmade things….You are truly in the nature, in the origin, in the untouched; no matter what weather.” (Male 25, 41 years, professional).

One participant stated the uniqueness of being at the top of the mountains and not in the valleys.

You are at the top and not in the valley. The moment when you stand on top and you see the sea of fog in the valley, this vast expanse it is just special” (Female 4, 24 years, semi-professional).

#### Friends (27)

The contribution of friends to the experience of freeriding was important to the participants and was mentioned by 66% (*n* = 27). Some participants (*n* = 14) described the value of friends in freeriding as a shared experience with people who experience the same passion. Others (*n* = 12) described the importance of shared trust in friends which was evoked through freeriding. They explained how they see freeriding not as a single sport because everybody has to rely on the partner(s) to have a higher survival chance in case of an avalanche. This mutual trust of being in a risk situation was mentioned and how it formed unique and deep friendships.

Definitely it is skiing with friends, because skiing is no single sport….When I go skiing with my friends somewhere then I have to trust my friends and my friends have to trust me. For me it is not just about skiing itself it is about who I go skiing with (Male 11, 23 years, semi-professional).

But I think that it is the shared experience with people who already share your fascination with the sport. You get closer because you lay your life in the hands of the others if something happens.” (Female 3, 28 years, professional);

It builds up very intense friendships which get closer through freeriding and are filled with memories (Male 3, 30 years, semi-professional).

Freeriding showed participants who they could trust and whom not. They got to know people better and stated how they only chose to ski with people they trusted and felt comfortable with. This meant that the participants were also aware of group dynamics and with whom they could reasonably discuss risk situations with. Understanding group dynamics appeared to be an important part of participants' risk-management strategies,

There are situations when I have a bad feeling and my partner has a good feeling then I ride it anyway although I wouldn't have if I had been alone, because I think my judgement isn't necessarily right. Then I trust the other person that my feeling might be wrong. But actually, that is only with one person, I don't trust most of the other people. There I trust myself more [laughs] (Male 7, 31 years, professional).

Another important factor when deciding who to ski with was, individual. This shows how the friends play a role even in preparation for the activity,

Who I go skiing with plays an important role. Because when I do exciting runs I need the right partner to do it, I can't do that with just anyone. They have to be like-minded people and people who I get along with really well. Then I can do more extreme things and on some days, we'll do mellow things. Those friendships grow more intense through that experience and are more valuable than those you'll make when drinking coffee for example. They are like-minded people who go on an adventure with me and they are really close friends (Male 25, 41 years, professional).

#### Balance (26)

*Balance* (*n* = 26; 65%) described how freeriding functions as a counterbalance to everyday life. Some participants (*n* = 16) described the activity of freeriding as crucial for their well-being but could not explain why it was crucial. Two participants reported to have or have had withdrawal symptoms when not participating in freeriding. They just realized that when they did not participate in their sport, they felt sick and without any purpose in life. The sport of freeriding gave them a direction in life and was also part of their personality,

I just had an inflammation in both knees and then you wake up in the morning and you are nothing, really nothing. If you can't ski and you can't do what you normally do, then you wake up and you would rather go back to sleep. The sport is extremely important for me that I am who I am, otherwise I am just really angry (Male 10, 23 years, semi-professional).

The impact of freeriding on personality was mentioned by participants in a number of different contexts, including in their understanding risk in their sport and how they were perceived by their families,

Skiing is a big part of us and would you let it be, then you would change yourself. And the relatives probably know that. But you shouldn't act recklessly (Male 13, 29 years, professional).

“Being in the moment” (*n* = 9) was a counterbalance to everyday life too. Not having to think about anything other than what they are doing at that exact moment in time, in some ways similar to meditating. This allowed participants to concentrate on simple things and to be away from the hustle and bustle of modern society. This feeling of being in the moment and away from modern society was facilitated by being on top of the mountain and having some geographic space between themselves and the stresses of society. Participants described how skiing calmed them down, leaving them more at ease and relaxed. Suggesting that freeriding may benefit participants in their day to day lives,

Freeriding has helped me in a lot of situations to conquer problems in everyday life, because those problems are not important up there anymore. It is just: “Am I doing it right or not?” “Should I ski this line or not?” Those are really reduced problems. It is like turning off the time (Female 2, 24 years, semi-professional);

For me freeriding is my balance to everyday life. In the mountains, I know my way. There you are away from the valley and the trouble and you just have this calmness. I love to go up there (Male 16, 25 years, semi-professional);

When I go in the mountains, I take my mobile with me, but I leave all worries, sorrows, and things I have to do at home. When I am back home from skiing, it's like having pressed the reset button. This is the time I have for myself (Male 21, 34 years, semi-professional);

Freeriding is such a complex sport. You don't have time to think about anything else. You're not allowed to make any faults. That's why you are forced to be focused and concentrated and completely in the moment (Female 3, 28 years, professional);

Skiing calms you down, you can reset, you are in the moment without thinking ahead or in the past. Everyone is stressed. If you go into the city you have to make sure that nobody runs you over. But it's like that, this is the pace of the society (Female 13, 28 years, professional).

#### Freedom/pleasure (26)

Freedom seemed to be an important part of freeriding to many participants; 65% (*n* = 26) of participants spoke about the lack of rules and restrictions in freeriding and the importance of that to them. In ski resorts there are rules, boundaries, and often a lot of other people to watch out for. But outside of this restrictive area, participants felt that they had the freedom to decide what to do and where to go. With this freedom comes responsibility and freeriders had to take charge of their own actions,

In the mountains, you can decide what to do; you decide the pace, whether it's going to be high, fast, steep, mellow. You decide (Female 13, 28 years, professional);

There are no rules and restrictions, you don't have to ride blue or red, you don't have to train always and be fast. It is free in freeriding; you can do whatever you want. And if you don't think about contests there are no comparisons, everybody has their own style (Female 5, 27 years, semi-professional).

Participants (*n* = 13) also talked about how they could be themselves and be creative through freeriding. Being creative, could involve developing an individual style while freeriding or looking for creative lines to ski,

I can live it up through skiing. To do what I want combined with the feeling that it feels great—self-expression with my own style without any rules (Male 5, 19 years, semi-professional);

I am myself up there [in the mountains] and just the freedom of it (Female 2, 24 years, semi-professional).

A number of participants (*n* = 4) said that freeriding was a sport that never got boring; because there are never ending possibilities to learn something new. Some participants also felt that what they learnt from their freeriding experiences helped them in their everyday lives,

It's a forever growing process and you can learn something every day. And also life-lessons for sure (Female 11, 27 years, semi-professional).

Some participants (*n* = 4) felt that it was cliché to say that they went freeriding to feel free, as “free” is in the name but nonetheless felt that this was an important part of it for them,

…and it is this cliché feeling of freedom—that I am not sitting in my flat somewhere and watch TV the whole day; that is no life content for me (Female 6, 30 years, semi-professional).

The description of freeriders (*n* = 9) to ride untracked snow and feeling this pleasure of riding was also coded to the motive of freedom because it necessarily involved the freedom of the terrain. Inside the resort on groomed slopes you will not find the snow what the participants were talking about.

Skiing for me is this unbelievable ease. Gliding through the snow; you kind of float down the mountain without a lot of effort. Doing jumps of 10 meters without so much strength (Male 24, 27 years, semi-professional).

…the feeling of snow under your feet. To experience that feeling of snow under your feet when you do fast powder turns. (Male 17, 26 years, semi-professional).

#### Habit (13)

It was categorized as a realization of having always done the sport and could be explained like an acquired behavioral habit. Whereas, *Habit* was described neutral, all other five themes were seen as positive effects in freeriding.

Some participants (*n* = 13) spoke about skiing being something that their lives evolved around and how freeriding became habitual. They grew up skiing and were good at it, so never tried something different. Unlike the other five themes they did not see the habit of skiing as having a positive effect but nor did they see it as detrimental. Participants felt comfortable and happy with skiing and through freeriding they could participate in a new way of skiing,

For me it's never been something I had to consider. Skiing was always normal for me and my favorite sport, maybe I also like playing football and surfing but like skiing I couldn't imagine doing anything else or anything else being more important to me (Male 20, 31 years, professional).

I don't know anything else [laughs] (Male 13, 29 years, professional).

### Aspects of risk in freeriding

**Figure 2:**
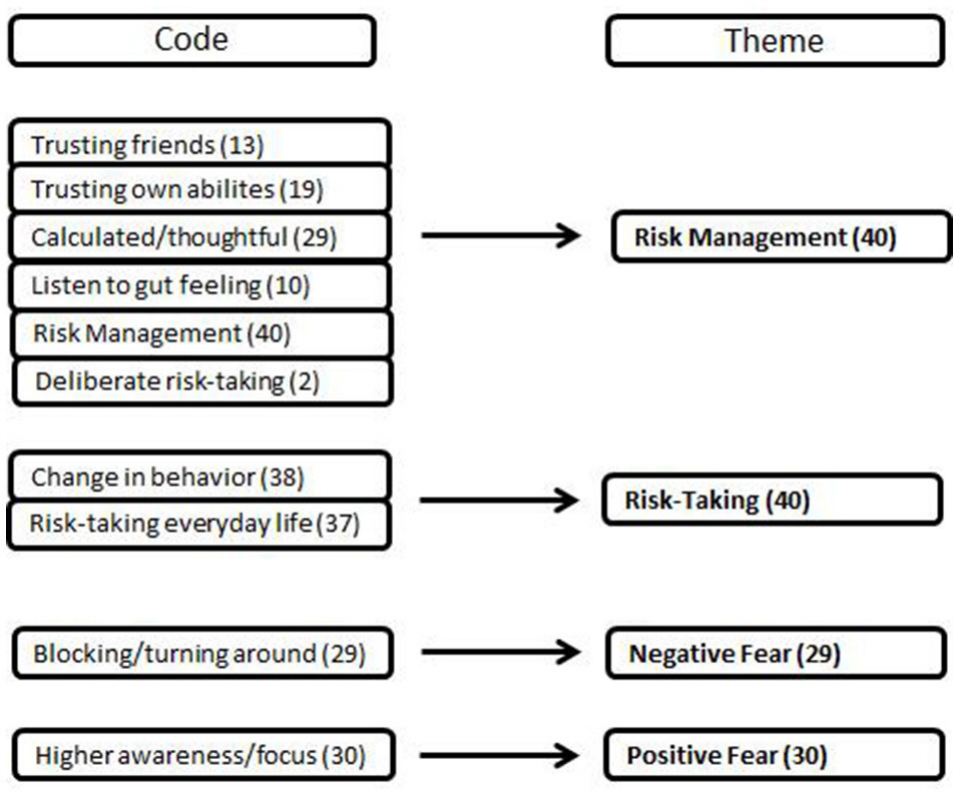
Codes and Themes of aspects of risk in freeriding. Codes were only accounted as one vote per person. Thus, the codes do not necessarily sum up to the according theme.

Participants reported aspects of risk in freeriding such as risk-taking, risk management as well as different experiences of fear (Figure 2). χ^2^-tests revealed no significant differences (*p* > 0.05) in risk aspects regarding gender or age. Both male and female participants described themselves to have a thoughtful and calculated risk management, experienced the same change in behavior through either external or own accidents or close calls. Both cohorts also based decisions on their gut feeling and trusted their abilities. The same could be seen for the age below 25 and above 25 years.

#### Risk-taking

Risk-taking was seen differently by participants depending who they compared themselves with. Some participants felt that they took more deliberate risks than the general public and that this was an inherent part of freeriding. However, when comparing themselves with other freeriders, most of the participants said that they do not take deliberate risks.

I think I take a really high risk. You are not stronger than nature. In the backcountry, no matter how much knowledge and ability you have, there is always the chance of a serious crash, or avalanches, or changes in the weather. I think the risk is really high because you are exposed to nature (Female 4, 24 years, semi-professional);

If you ask the general public if I am risk-loving, they'll certainly say yes. If you ask me I'll say no. I would never take a risk which I don't stand up for. Out of my view I'd say no, I am not risk-loving, but I have already invested a lot in the sport and can test my limits at a higher level. Thoughts about “am I the worst daredevil” have no place inside me (Male 3, 30 years, semi-professional).

When talking about risk-taking in everyday life, 17 participants said that they look for challenges in everyday life, but never take unnecessary risks. In the context of driving, 12 participants said that they do not speed because other people are at risk, whilst three participants said that they like to speed while driving and do not always follow the rules of the road. Most participants reported how they only take risks when they have an influence on the outcome, these participants felt that driving was more dangerous than freeriding and a less predictable outcome.

Eight participants said that freeriding gave them a better understanding of risk in other areas of their life. As one participant explained how the confrontation with risk in freeriding helped him to increase awareness in everyday life.

I think when you are confronted with risk and the consequences of it you might get a higher awareness of what can happen and how you avoid it, for example while driving a car. I think to confront oneself with risk and the consequences of it, is safer than just throwing yourself into something without even knowing what could happen (Male 24, 27 years, semi-professional).

Nearly all participants reported changes in their risk-taking over time, either due to being involved in a freeriding accident or close call (*n* = 23) (close call as defined by Woodman et al. (2010, p. 480): “Close calls are incidents that come very close to resulting in a negative outcome but that fail to materialize into a negative outcome. As such, close calls are largely the same as an accident except for the outcome.”), or through an external accident/fatality (*n* = 14) or simply through experience (*n* = 14).

#### Risk management

The use of risk management strategies appeared to be an indispensable part of freeriding; all participants reported using risk management strategies, most described their actions as calculated and thoughtful (*n* = 29). Only two participants felt that their behavior could be described as reckless. One participant said that saying “no” to something was never an option, and that taking risks with the life was common as the risk of dying was accepted. The participant also felt that this was unlikely to change until a major accident happened. Another participant who was categorized as a deliberate risk-taker said that when there was a lot of snow (i.e., higher risk) he turned his head off but afterwards he would think about the things that could have gone wrong. Most participants described a risk-management strategy which followed a calculation strategy. Risk management involved a number of factors: information gathered prior to participation (e.g., weather forecast, snow conditions, terrain, etc.); participants' experience; group dynamics; and the current local situation. One participant explained this process really clearly,

My [risk] assessment is based on my experience and on the information that I have. If I am in the backcountry, the steeper it gets the more components I have to consider and the more components have to turn green to be able to do it… So, the decision should be taken by the logic. This is a process. Is it basically possible and then you're considering the actual situation: “is it safe given the snow conditions?” This is how I make a decision. It is a rational thing. I always try to minimize it to an acceptable risk, but where this acceptable risk is, is my decision. You depend on your knowledge, your experience and your instinct (Male 3, 30 years, semi-professional).

#### Fear

Participants distinguished between positive and negative fear. Positive fear led to higher awareness, focus, and concentration whereas negative fear blocked actions.

And when you start to panic, I've never really panicked but maybe it went a little bit in that direction from time to time, then everything is blocked—finished (Male 7, 31 years, professional).

Many participants tried to avoid experiencing negative fear (*n* = 26), when they felt that they might experience it, they turned around or made an alternate plan. Positive fear was seen as being aware of the dangers of freeriding and most participants (*n* = 29) saw this positive fear as something that protected them, as most injuries occurred when participants felt that they were not fully concentrated.

It [fear] makes you hyperaware of everything around you, it makes your mind work faster you can consider things you otherwise wouldn't think about; it keeps you safe in a way, cause if you don't have fear you gonna do stupid things (Male 20, 31 years, professional).

## Discussion

The present study sought to identify the motives for participation in a single high-risk sport, freeriding. This inquiry revealed five main themes and one subtheme of motivation. All five of the main themes positively contributed to the experience of freeriding. Habit was classed as a sub-theme because only 13 participants spoke about it. Participants' acknowledgment that they had been very focused on skiing since the beginning was an important part of *Habit*. It was always a main part of their life and they never really considered the motives of participation because it was always their way of living or “normal” to practice it. Unlike the other themes, *Habit* was seen as neutral rather than positive or negative. Participants reported to have been skiing their whole life and all participants who named *Habit* as a motive had a background in ski racing. Since all of those participants started at a young age with skiing and racing, freeriding might have been their first opportunity to take part in a skiing discipline of their own choice. Participants saw starting freeriding as a “natural progression,” rather than a deliberate choice; they could continue doing what they were good at (skiing) but had a new way of performing.

Deliberately seeking high-risk situations did not emerge as a motive for freeriding, but the challenge of developing risk-management strategies was seen as an integral part of the activity. All participants said that they were well aware of the dangers of freeriding and only one of the 40 participants reported taking deliberate risks. One participant explained that she knows the inherent risks in freeriding are part of the experience of freeriding and that it “gives you the adrenaline” (Female 11, 27 years, semi-professional). Whilst this could be considered evidence for sensation seeking, it was only evident in one participant's transcript, thus it was treated as a “side effect” in the present analyses. Since freeriders reported high preparation times for the activity without the presence of intense sensations, the theme *Challenge* comprises a more complex motivation which is about the mastering of the challenge rather than seeking sensations. Some participants differentiated their risk-taking between big mountain freeriding and cliff jumping in a more controlled environment. Whilst most participants stated that they generally choose to stay within their technical abilities, they were more likely to push their limits when jumping in a controlled environment where the risk was lower rather than when skiing in an exposed environment. Nevertheless, they reported to be well aware of their capabilities and only chose tasks which they knew they could manage. Participants were well aware of the risky nature of the sport and used calculated risk-management strategies. Factors incorporated in their calculations included, planning and preparation, the choice of partner(s) for the activity, the belief in oneself and one's abilities, experience, knowledge, and the seriousness of the situation. Whilst the dangerous nature of the sport contributed to participant's experience, none of them reported actively seeking dangerous situations. Minimizing the risk as much as possible was a goal named by 39 of the 40 participants. This suggests that the traditional “sensation seeking” explanation (Diehm and Armatas, 2004; Zuckerman, 2008) for participation in high-risk sport is not suitable for freeriding. Though the theme *Challenge* was the most named motive in participation, it was the challenge of avoiding rather than seeking life-threatening situations.

The theme *Challenge* was also identified in an hermeneutic approach investigating a mixed sample of high-risk sport participants (Willig, 2008), whereas that theme mostly included the code “going to the limits.” The challenge with the environment and the “mind game” was not explicitly stated by Willig (2008) but seemed to play an important role for the participants of the present study. Brymer and Schweitzer (2013b) described how participants acknowledged the power of nature (“There is an appreciation that the natural world is much more powerful than the self” (Brymer and Schweitzer, 2013b, p. 871). Participants in this research also felt that it was important to respect the natural environment and also felt that this “positive fear” helped to keep them safe as it made them concentrate on the task at hand, thus making them less likely to have an accident or be injured. However, too much fear or “negative fear” led to situations where participants reported turning away from their objective. It is possible that “positive fear” represents a state of optimal arousal and that “negative fear” represents over arousal with accidents being more likely when participants are under aroused and therefore not concentrating fully (Yerkes and Dodson, 1908). Some participants reported their arousal comparable to the catastrophic curve (Hardy and Parfitt, 1991), with participants reporting their actions being blocked when they have too much fear. Participants understood the power of nature, stating that there is always a chance that something could go wrong. They also understood that even if you are highly experienced you still need to prepare fully as “*The mountain knows no conscience*” (Male 21, 34 years, semi-professional). Some participants lost friends or experienced their own close calls, following these events, they changed their behavior. Others said that their behavior was constantly evolving, changing with every new experience. This is comparable with the findings of Willig (2008) who stated (p. 695) “The experience of extreme sport, then, involves reflection and monitoring of one's developing capabilities.” Participants stated that they reflected on their experiences and that negative experiences were more likely to lead to a change in behavior. Interestingly, changes in behavior were related to experience not age. This means that at least in freeriding and maybe in other high-risk sports which involve knowledge and skills, the belief that younger people will be more risky might not hold true. Freeriding might teach also younger people after some years of practice to make educated choices about risk situations.

There are some similarities between the results of this study and those of Willig (2008) however, some of the motives for freeriding were not uncovered in Willig's (Willig, 2008) study of a mixed group of high-risk sports participants. Both studies showed that other people played a role in participants' motives for taking part in their sport. In Willig's (Willig, 2008) investigation the presence, or absence, of people was important in setting the *context* for participants whilst in this study freeriders said that it was very important that their friends contributed to their experience. In this study, the contribution of friends to the experience was either mentioned as a shared experience with like-minded people, or as a shared trust between friends in freeriding. It was reported that because of this shared trust, freeriding helped to develop deep friendships. This was illustrated by a participant as following:

Those friendships grow more intense through that experience and are more valuable than those you'll make when drinking coffee for example. They are like-minded people who go on an adventure with me and they are really close friends (Male 25, 41 years, professional).

Having trusted partners in freeriding is also part of their reported risk-management. In avalanche accidents, it is usually a member of the victims group, if not the victim themselves, who triggers the avalanche (Zweifel and Haegeli, 2014). Furthermore, participants knew that having a partner who is able to rescue them from an avalanche quickly is crucial for survival, as highest survival rates were found in victims who were rescued within the first 15 min of burial time (Procter et al., 2016). Thus, it is important that freeriders choose the right partner(s); participants in this study reported this as part of their risk-management strategies. Sometimes it was only after an accident or close call that participants realized who they could actually trust. Being involved in an accident or close call made participants more careful about who they went freeriding with.

Previous research has not explicitly identified *Friends* as a motive for participation in high-risk sports, nor as a factor in risk-management, however this research has clearly demonstrated the role of friends in both. One explanation for this could be that the present research used athletes from a single sport, freeriding, which some participants described as a “*social sport*” differentiating it from other sports where people competed against one another. Willig (2008) noted that differences in social aspects were seen between sport types. Whereas, the skydiver focused more on camaraderie, the mountaineer focused more on the flow experience.

Being in nature was another motive for freeriding. Brymer and Gray (2010) described the importance of nature to the high-risk sport participants. “Participants seem clear that extreme sport participation provided a context for appreciating humanity's connection to the natural world and the realization that humanity is just a small part of the greater whole” (Brymer and Schweitzer, 2013b, p. 371). The findings of the present research support these studies. The vast majority of high-risk sports are practiced outdoors in nature and participants described how they value the untouched nature. Thus, one might assume that they gain a richer understanding of environmental factors and might further try to protect their environment which is crucial for participating in freeriding (“*and taking care of the nature. For me it's important to be clean*.” Male 6, 24 years, semi-professional). Being in the outdoors, in areas of nature untouched by humans, might also serve as a counterbalance to the built environment which is present in everyday life.

Having this counterbalance was important to participants; it was further shown by the theme *Balance*. “Being in the present” was one aspect of *Balance* as concentrating on the task at hand allowed participants to forget about day-to-day stresses. Similarly, forgetting problems in everyday lives was reported as a positive effect of participation by Big-Wave Surfers in California (Wiersma, 2014). “Being in the present” was also discussed by Willig (2008), she showed that regular participation in high-risk sports was therapeutic, and reduced stress levels and concerns. Participants in this study described how freeriding helped them to cope with their lives, and how they felt like they had lost something if they could not participate in their sport (e.g., due to an injury). This suggests that freeriding provided some regulatory benefits to participants that transfers to the rest of their lives. Other research has shown that high-risk sports can help participants deal with their day-to-day lives (Woodman et al., 2010; Barlow et al., 2013).

Contrary to findings of previous studies, participants in the present study did not report being obsessed with freeriding. Whilst withdrawal symptoms have been reported by climbers when not participating (Heirene et al., 2016) only two participants reported them in this study. The seasonal nature of skiing may prevent participants forming the same attachments with the activity as participants of perennial activities do, in addition to this participants said that they practice different summer sports, namely mountain sports which could be performed in the surrounding environment (e.g., mountain biking, climbing, mountaineering, paragliding etc.). The same number of participants named the motives *Balance* and *Freedom/Pleasure* (*n* = 26). Freeriding provided participants in this study with an opportunity to experience freedom as they could decide: where to ski, how they skied, and who they skied with. This sense of freedom might also be described as a sense of agency. Experiencing agency has been identified as a motive for participation in mountaineering (Barlow et al., 2013). Having no restrictions meant to be responsible for the own actions. similar to the theme *freedom as choice and responsibility* identified by Brymer and Schweitzer (2013b).

This research is the first qualitative inquiry to examine the effect of age on the motives for participating in a high-risk sport and no age differences emerged from the data. Other studies have controlled for age by limiting the age range of their sample (e.g., over 30 years old) as it has been reported that younger people (16–25 years) search for opportunities to take deliberate risks (see Brymer and Schweitzer, 2013b). The present research suggests that experience may be a more important factor than age in predicting motives for freeriding in adults (i.e., over 18 years). It is important to note that the age group used in the study is not entirely congruent with that of younger people (16–25 years), it remains unclear how the current findings relate to adolescents. Future research should examine the relationship between age, experience, and motivation for participation in high-risk sport.

χ^2^-tests did not reveal any gender or age differences in motives for freeriding or in aspects of risk-taking and management in this sample of experienced freeriders. There are a number of possible reasons that no differences were found, it may be that the groups were too small, however there are a number of other possible explanations. It is possible that the higher fatality rates in men (Jekich et al., 2016) could be explained by gender differences in participation (Procter et al., 2014) rather than differences in risk-taking between men and women. This investigation only evaluated highly experienced participants and the variation in experience might not have been large enough to detect differences in risk-taking and management due to differences in experience. Raue et al. (2015) found differences in risk perceptions over time between experienced and less experienced ski tourers. Findings of the present analysis should not be generalized to less experienced freeriders, however, a logical extension of this study would be to repeat the study but with freeriders who had different levels of experience and to compare their motives with those of experienced participants.

### Strengths, limitations, and directions for future research

Qualitative research is often used to examine issues in great detail and depth (Anderson, 2010). Using an inductive approach, as in this study, allows researchers to uncover factors that would go unnoticed in a deductive approach (Anderson, 2010). Two important strengths of this study are that it both examined a single sport and had a much higher sample size than many previous qualitative studies in high-risk sport (Willig, 2008; Kerr and Houge Mackenzie, 2012; Brymer and Schweitzer, 2013b; Jones et al., 2017). In addition this study included participants with a range of ages, gender, and profession levels, something few previous studies have done. However, readers should avoid generalizing the results of this study beyond highly experienced freeriders as it has been shown repeatedly that people have different motives for participating in different high-risk sports.

χ^2^-tests were carried out with a relatively small sample and as such may be underpowered. Therefore, one obvious direction for future research within the freeride skiing population would be to better understand the relationships, or lack thereof, between age and gender with motives for participation and risk-taking.

Furthermore, research should consider the effect of experience on these relationships. In the wider high-risk sport population, further research should, as in this study, try to identify motives for participation in individual high-risk sports, which have remained hidden in analyses of heterogeneous populations. Researchers could also consider the influence of the environment and duration of the activity on this.

## Conclusion

This study has shown that freeriders experience several positive effects of freeriding. Challenging oneself, experiencing nature, building deep friendships, a counterbalance to everyday life, and escape from restrictions were driving motivations named for participation in freeriding. Participation was not driven by a desire to seek out high-risk situations, but was about managing risk to an acceptable level thus, allowing participants to experience the benefits of freeriding. In examining a single high-risk sport (i.e., freeriding), two new motives for participation *(Friends* and *Habit)* were identified. Friends contributed to the experience of freeriding as like-minded people and as trusted partners from which deep friendships were reported. *Habit*, was characterized by the view that skiing had always been a part of their life and possibly not something that they had made a conscious decision to do with freeriding being a “natural progression”. *Habit* held neither positive nor negative sentiment with participants and was only mentioned by 13 of the 40 participants. Analyses of the present data did not show any age or gender differences regarding motives of participation or aspects of risk in freeriding. This might indicate that experience and knowledge of the sport are much more important than age or gender. Some motives were similar to prior qualitative research in high-risk sports. Future research should evaluate further high-risk sport participants of different terrain based activities and compare their motives and aspects of risk and might implement those motives in quantitative research.

## Author contributions

AF: Contributed to the conception of the research and study design. Collected the data, transcibed and analyzed. Wrote the draft of the manuscript in full. WH: Rewrote large sections, questionned and discussed the outcome and interpretation of the data. DP and F-GH: Collected and transcribed the data. MK: Contributed to the conception of the research and study design. Supervised the work and acted as a critical friend in all stages of the anlysis of data.

### Conflict of interest statement

The authors declare that the research was conducted in the absence of any commercial or financial relationships that could be construed as a potential conflict of interest.

## Footnotes

^1^MAXQDA: software for qualitative data analysis (1989–2017). Berlin, Germany: VERBI Software—Consult—Sozialforschung GmbH.

## References

[B1] Anderson C. (2010). Presenting and evaluating qualitative research. Am. J. Pharm. Educ. 74:141. 10.5688/aj7408141 21179252 PMC2987281

[B2] Barlow M. Woodman T. Chapman C. Milton M. Stone D. Dodds T.. (2015). Who takes risks in high-risk sport? The role of alexithymia. J. Sport Exerc. Psychol. 37, 83–96. 10.1123/jsep.2014-0130 25730894

[B3] Barlow M. Woodman T. Hardy L. (2013). Great expectations: different high-risk activities satisfy different motives. J. Pers. Soc. Psychol. 105, 458–475. 10.1037/a0033542 23795909

[B4] Breivik G. (1999). Empirical Studies of Risk Sport. Oslo: Norges Idrettshøgskole. Institutt for samfunnsfag.

[B5] Brugger H. Durrer B. Elsensohn F. Paal P. Strapazzon G. Winterberger E.. (2013). Resuscitation of avalanche victims: evidence-based guidelines of the international commission for mountain emergency medicine (ICAR MEDCOM): intended for physicians and other advanced life support personnel. Resuscitation 84, 539–546. 10.1016/j.resuscitation.2012.10.020 23123559

[B6] Brymer E. (2010). Risk taking in extreme sports. A Phenomenol. Perspect. Ann. Leisure Res. 13, 218–238. 10.1080/11745398.2010.9686845

[B7] Brymer E. Gray T. (2010). Developing an intimate relationship with nature through extreme sports participation. Leisure/Loisir 34, 361–374. 10.1080/14927713.2010.542888

[B8] Brymer E. Oades L. G. (2009). Extreme sports. A positive transformation in courage and humility. J. Hum. Psychol. 49, 114–126. 10.1177/0022167808326199

[B9] Brymer E. Schweitzer R. (2013a). Extreme sports are good for your health: a phenomenological understanding of fear and anxiety in extreme sport. J. Heal. Psychol. 18, 477–487. 10.1177/1359105312446770 22689592

[B10] Brymer E. Schweitzer R. (2013b). The search for freedom in extreme sports: a phenomenological exploration. Psychol. Sport Exerc. 14, 865–873. 10.1016/j.psychsport.2013.07.004

[B11] Castanier C. Le Scanff C. Woodman T. (2010). Who takes risks in high-risk sports? A typological personality approach. Res. Q. Exerc. Sport 81, 478–484. 10.1080/02701367.2010.10599709 21268472

[B12] Castanier C. Le Scanff C. Woodman T. (2011). Mountaineering as affect regulation: the moderating role of self-regulation strategies. Anxiety Stress Coping 24, 75–89. 10.1080/10615801003774210 20397078

[B13] Cazenave N. Le Scanff C. Woodman T. (2007). Psychological profiles and emotional regulation characteristics of women engaged in risk-taking sports. Anxiety Stress Coping 20, 421–435. 10.1080/10615800701330176 17999241

[B14] Diehm R. Armatas C. (2004). Surfing. An avenue for socially acceptable risk-taking, satisfying needs for sensation seeking and experience seeking. Pers. Indiv. Differ. 36, 663–677. 10.1016/S0191-8869(03)00124-7

[B15] Elliott R. Fischer C. T. Rennie D. L. (1999). Evolving guidelines for publication of qualitative research studies in psychology and related fields. B. J. Clin. Psychol. 38, 215–229. 10.1348/014466599162782 10532145

[B16] Ewert A. Gilbertson K. Luo Y.-C. Voight A. (2013). Beyond because it's there. Motivations for pursuing adventure recreational activities. J. Leisure Res. 45, 91 10.18666/JLR-2013-V45-I1-2944

[B17] Haegeli P. Gunn M. Haider W. (2012). Identifying a high-risk cohort in a complex and dynamic risk environment: out-of-bounds skiing - An example from avalanche safety. Preven. Sci. 13, 562–573. 10.1007/s11121-012-0282-5. 22961005

[B18] Haegeli P. Pröbstl-Haider U. (2016). Research on personal risk in outdoor recreation and nature-based tourism. J. Outdoor Recreat. Tour. 13, 1–9. 10.1016/j.jort.2016.02.001

[B19] Hardy L. Parfitt G. (1991). A catastrophe model of anxiety and performance. B. J. Psychol. 82, 163–178. 10.1111/j.2044-8295.1991.tb02391.x 1873650

[B20] Heggie T. W. Caine D. J. (2012). Epidemiology of injury in adventure and extreme sports. Basel: S. Karger AG. 10.1159/000338558 22824836

[B21] Heirene R. M. Shearer D. Roderique-Davies G. Mellalieu S. D. (2016). Addiction in extreme sports: an exploration of withdrawal states in rock climbers. J. Behav. Addict. 5, 332–341. 10.1556/2006.5.2016.039 27348554 PMC5387785

[B22] Jekich B. M. Drake B. D. Nacht J. Y. Nichols A. Ginde A. A. Davis C. B. (2016). Avalanche fatalities in the United States: a change in demographics. Wilderness Environ. Med. 27, 46–52. 10.1016/j.wem.2015.11.004 26948553

[B23] Jones G. Milligan J. Llewellyn D. Gledhill A. Johnson M. I. (2017). Motivational orientation and risk taking in elite winter climbers. A qualitative study. Int. J. Sport. Exerc. Psychol. 15, 25–40. 10.1080/1612197X.2015.1069876

[B24] Kerr J. H. Houge Mackenzie S. (2012). Multiple motives for participating in adventure sports. Psychol. Sport Exerc. 13, 649–657. 10.1016/j.psychsport.2012.04.002

[B25] Lafollie D. Le Scanff C. (2007). Détection des personnalités à risque dans les sports à sensations fortes Detection of high-risk personalities in risky sports. L'Encéphale 33, 135–141. 10.1016/S0013-7006(07)91543-2 17675908

[B26] Leiter A. M. Rheinberger C. M. (2016). Risky sports and the value of safety information. J. Econ. Behav. Organ. 131, 328–345. 10.1016/j.jebo.2016.09.003

[B27] Llewellyn D. J. Sanchez X. (2008). Individual differences and risk taking in rock climbing. Psychol. Sport Exerc. 9, 413–426. 10.1016/j.psychsport.2007.07.003

[B28] Pain M. T. Pain M. A. (2005). Essay risk taking in sport. Lancet 366, S33–S34. 10.1016/S0140-6736(05)67838-5 16360743

[B29] Paquette L. Lacourse É. Bergeron J. (2009). Construction d'une échelle de prise de risques et validation auprès d'adolescents pratiquant un sport alpin de glisse [Construction and validation of a risk-taking scale for adolescent practitioners of Alpine ski sports]. Canad. J. Behav. Sci. 41, 133–142. 10.1037/a0015256

[B30] Patton M. Q. (1990). Qualitative Research & Evaluation Methods. London: SAGE Publications, Inc.

[B31] Procter E. Strapazzon G. Dal Cappello T. Castlunger L. Staffler H. P. Brugger H. (2014). Adherence of backcountry winter recreationists to avalanche prevention and safety practices in northern Italy. Scand. J. Med. Sci. Sports 24, 823–829. 10.1111/sms.12094 23815413

[B32] Procter E. Strapazzon G. Dal Cappello T. Zweifel B. Würtele A. Renner A.. (2016). Burial duration, depth and air pocket explain avalanche survival patterns in Austria and Switzerland. Resuscitation 105, 173–176. 10.1016/j.resuscitation.2016.06.001 27312137

[B33] Raue M. Streicher B. Lermer E. Frey D. (2015). Being active when judging risks. Bodily states interfere with accurate risk analysis. J. Risk Res. 20, 445–462. 10.1080/13669877.2015.1057206

[B34] Reynier V. Vermeir K. Soule B. (2014). Social representations of risks among winter sports participants. A focus on the influence of sports practice and style in the French Alps. Sport Soc. 17, 736–756. 10.1080/17430437.2014.882904

[B35] Ryan R. M. Weinstein N. Bernstein J. Brown K. W. Mistretta L. Gagné M. (2010). Vitalizing effects of being outdoors and in nature. J. Environ. Psychol. 30, 159–168. 10.1016/j.jenvp.2009.10.009

[B36] Smith B. Sparkes A. C. (2009). Narrative analysis and sport and exercise psychology. Understanding lives in diverse ways. Psychol. Sport. Exerc. 10, 279–288. 10.1016/j.psychsport.2008.07.012

[B37] Sparkes A. C. Smith B. (2014). Qualitative Research Methods in sport, Exercise and Health: From Process to Product. Abingdon: Routledge.

[B38] Techel F. Zweifel B. Winkler K. (2015). Analysis of avalanche risk factors in backcountry terrain based on usage frequency and accident data in Switzerland. Nat. Hazards Earth Syst.Sci. 15, 1985–1997. 10.5194/nhess-15-1985-2015

[B39] Vargyas G. (2016). Backcountry skiers, avalanche trauma mortality, and helmet use. Wilderness Environ. Med. 27, 181–182. 10.1016/j.wem.2015.09.020 26712332

[B40] Wiersma L. D. (2014). A phenomenological investigation of the psychology of big-wave surfing at Maverick's. Sport Psychol. 28, 151–163. 10.1123/tsp.2013-0001

[B41] Willig C. (2008). A phenomenological investigation of the experience of taking part in ‘extreme sports.’ J. Heal. Psychol. 13, 690–702. 10.1177/1359105307082459 18519442

[B42] Woodman T. Barlow M. Bandura C. Hill M. Kupciw D. Macgregor A. (2013). Not all risks are equal: the risk taking inventory for high-risk sports. J. Sport Exerc. Psychol. 35, 479–492. 10.1123/jsep.35.5.479 24197716

[B43] Woodman T. Hardy L. Barlow M. Le Scanff C. (2010). Motives for participation in prolonged engagement high-risk sports. An agentic emotion regulation perspective. Psychol. Sport Exerc. 11, 345–352. 10.1016/j.psychsport.2010.04.002

[B44] Woodman T. Huggins M. Le Scanff C. Cazenave N. (2009). Alexithymia determines the anxiety experienced in skydiving. J. Affect. Disord. 116, 134–138. 10.1016/j.jad.2008.11.022 19103465

[B45] Yerkes R. M. Dodson J. D. (1908). The relation of strength of stimulus to rapidity of habit-formation. J. Comp. Neurol. Psychol 18, 459–482. 10.1002/cne.920180503

[B46] Zuckerman M. (2008). Sensation Seeking and Risky Behavior. 2. print. Washington, DC: American Psychological Association.

[B47] Zuckerman M. Neeb M. (1979). Sensation seeking and psychopathology. Psychiatry Res. 1, 255–264. 10.1016/0165-1781(79)90007-6 298353

[B48] Zweifel B. Haegeli P. (2014). A qualitative analysis of group formation, leadership and decision making in recreation groups traveling in avalanche terrain. J. Outdoor Recreat. Tour. 5, 17–26. 10.1016/j.jort.2014.03.001

