# Supplementary material for: A Qualitative Approach on Motives and Aspects of Risks in Freeriding
Source: Front Psychol. 2017 Nov 14;8:1998. doi: 10.3389/fpsyg.2017.01998 (PMC5694463; doi:10.3389/fpsyg.2017.01998)
Supplement: Supplementary file 1 [file Table1.docx]

Table 1. Cross table of motives and gender

| Motives | female | male | Significance |
| --- | --- | --- | --- |
| BALANCE | 9 (69%) | 17 (63%) | p = 1.0^a^ |
| FREEDOM/PLEASURE | 7 (54%) | 20 (74%) | p = .16^a^ |
| CHALLENGE | 12 (92%) | 23 (85%) | p = 1.0^a^ |
| NATURE | 10 (77%) | 21 (78%) | p = 1.0^a^ |
| FRIENDS | 8 (62%) | 19 (70 %) | p = .72^a^ |
| HABIT | 3 (23%) | 10 (37%) | p = 1.0^a^ |
| N | 13 | 27 |  |

^a^Not all data met the requirements of an expected frequency above 5.
