# Supplementary material for: A Qualitative Approach on Motives and Aspects of Risks in Freeriding
Source: Front Psychol. 2017 Nov 14;8:1998. doi: 10.3389/fpsyg.2017.01998 (PMC5694463; doi:10.3389/fpsyg.2017.01998)
Supplement: Supplementary file 2 [file Table2.docx]

Table 2. Cross table of motives and age

|  | Age ≤ 25 | Age > 25 | Significance |
| --- | --- | --- | --- |
| BALANCE | 9 (75%) | 17 (61%) | p = .48 ^a^ |
| FREEDOM/PLEASURE | 8 (67%) | 19 (68%) | p = 1.0 ^a^ |
| CHALLENGE | 10 (83%) | 25 (89%) | p = .63^a^ |
| NATURE | 9 (75%) | 22 (79%) | p = 1.0^a^ |
| FRIENDS | 9 (75%) | 18 (64%) | p = .72 ^a^ |
| HABIT | 3 (25%) | 10 (36%) | p = .72 ^a^ |
| N | 12 | 28 |  |

^a^Not all data met the requirements of an expected frequency above 5.
